# Online Determination of Graphene Lattice Orientation Through Lateral Forces

**DOI:** 10.1186/s11671-016-1553-z

**Published:** 2016-08-02

**Authors:** Yu Zhang, Fanhua Yu, Guangyong Li, Lianqing Liu, Guangjie Liu, Zhiyong Zhang, Yuechao Wang, Uchechukwu C. Wejinya, Ning Xi

**Affiliations:** 1 Department of Computer Science and Technology, Changchun Normal University, Changchun, 130032 China; 2 State Key Laboratory of Robotics, Shenyang Institute of Automation Chinese Academy of Sciences, Shenyang, 110016 China; 3 Department of Electrical and Computer Engineering, University of Pittsburgh, Pittsburgh, 15261 USA; 4 Department of Mechanical Engineering, University of Arkansas, Fayetteville, AR 72701 USA; 5 Emerging Technologies Institute, The University of Hong Kong, Hong Kong, China

**Keywords:** Graphene, Lattice orientation, Manufacturing, Frequency

## Abstract

Rapid progress in graphene engineering has called for a simple and effective method to determine the lattice orientation on graphene before tailoring graphene to the desired edge structures and shapes. In this work, a wavelet transform-based frequency identification method is developed to distinguish the lattice orientation of graphene. The lattice orientation is determined through the different distribution of the frequency power spectrum just from a single scan line. This method is proven both theoretically and experimentally to be useful and controllable. The results at the atomic scale show that the frequencies vary with the lattice orientation of graphene. Thus, an adjusted angle to the desired lattice orientation (zigzag or armchair) can easily be calculated based on the frequency obtained from the single scan line. Ultimately, these results will play a critical role in wafer-size graphene engineering and in the manufacturing of graphene-based nanodevices.

**Electronic supplementary material:**

The online version of this article (doi:10.1186/s11671-016-1553-z) contains supplementary material, which is available to authorized users.

## Background

Graphene has been known as the replacement for silicon due to its unique electronic, physical, and mechanical properties as well as its wide range of applications [1, 2]. Although graphene shows extraordinary performance in fast transistors [3–7], super-capacitor [8], and highly sensitive sensors [9–11], the absence of an energy bandgap is still a grand challenge for applications in semiconductor nanodevices. Fortunately, previous studies have shown that graphene nanoribbons can display metallic or semiconducting properties due to different edges (zigzag or armchair) with the bandgap tunable by the width [12–15].

To date, several different graphene patterning methods such as Catalytic Cutting Technique [16–19], SPM-based Electric Field Tailoring Technique [20–22], AFM Scratching Technique [23, 24], Photocatalytic Patterning Approach [25], and Energy Beam Cutting method [26–28] have been developed. But whatever the cutting technique is, one of the prerequisites for tailoring graphene into desired nanodevice is to know its original lattice orientation, based on which the desired geometry configuration can be designed. Moreover, recent progress has been made in making large area graphene, with the largest reports on 4-in. wafers (http://www.electronicsweekly.com/Articles/2010/02/03/47937/100mm-graphene-wafer-grown.htm). Therefore, it becomes absolutely necessary to develop a simple, fast, flexible, and controllable method to determine the lattice orientation (zigzag and armchair) of wafer-size graphene on various substrates before manufacturing. Recently, Sasaki et al. reported friction anisotropy on graphene studied by molecular mechanics simulation. It revealed the possibility of identifying the lattice orientation on graphene theoretically [29]. Although AFM can image surface of material in atomic resolution [30, 31], the imaging conditions are very strict, especially in air under ambient conditions. The imaging process is easily affected by factors such as environment (including humidity, temperature, etc.) and probes. Additionally, the repeatability is very low. Even if the researchers who have rich atomic observation experiences, it also needs to take hours to obtain a stable and clear atomic resolution image.

In this paper, a wavelet transform-based frequency ratio identifying method is developed to determine the lattice orientation of graphene theoretically and experimentally. The uniqueness of the proposed method lies in using one or two friction scanning lines that can quickly distinguish graphene lattice orientation. Both theoretical and experimental results at the atomic scale have shown that the frequency ratio vary with the lattice orientation on graphene, based on which the lattice orientation on graphene can easily be distinguished. The findings in this paper will play a critical role in wafer-size graphene engineering and in the development of graphene-based nanodevices.

## Methods

The friction measurements on graphene were performed with a Multimode AFM (now Bruker) in air under ambient conditions (43 to 47 % relative humidity, 23 to 26 °C). AFM probes with rectangular cantilevers were used with scan size 5 nm across. Its radius, length, width, height, and thickness are 10, 450, 50, 10, and 2 μm, respectively. The normal spring constant is 0.2 N/m. The scan rate has to be more 10 Hz. The total number of lines per image, which defines the pixel resolution of the image, was kept constant at 256. All images, except otherwise indicated, were flattened with a first-order line-wise correction fit. Graphene (Additional file 1: Figure S1) was prepared by using micromechanical cleavage of bulk graphite [1]. Monolayer CVD graphene (Fig. 6) was transfer to an electrode chip by using bubbling transfer [32]. The electrodes were fabricated by standard photolithography and lift-off techniques. The morphology and structure of monolayer CVD graphene were characterized using an optical microscope (KH-7700, Hirox Inc.), AFM (Veeco Dimension 3100, tapping mode). The wavelet transforms [33, 34] were used here to obtain frequency information and signal filtering. It was performed with Daubechies wavelet (db9). Four-step wavelet decomposition (see Additional file 1: Figure S3) is chosen.

## Results and Discussion

Fiction measurements for different directions were performed by changing the scan angles (0°, 5°, 14°, 25°, 30°, 35°, 44°, 49°, and 55°, respectively. See Additional file 1: Figure S1). For the comparison of simulation and experiment results, the lattice angle 0° in experiments is defined as a zigzag orientation, which is in the anticlockwise direction nearest to the scanning direction. Thus 30° indicates an armchair orientation. The parameters used in the simulation were depicted in Additional file 1.

**Fig. 1:**
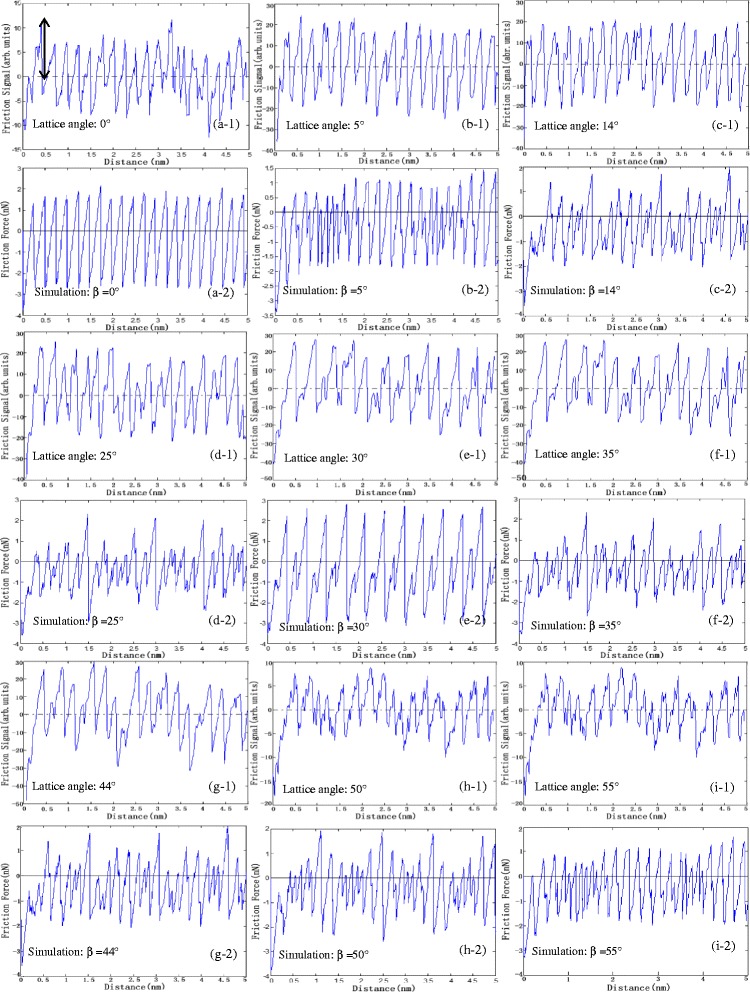
The single friction force signal profile in experiment and simulation results. *(x-1)* shows the experimental result, and *(x-2)* displays simulation result. The *vertical double-ended arrow* indicates a sliding height measured with respect to zero

**Fig. 2:**
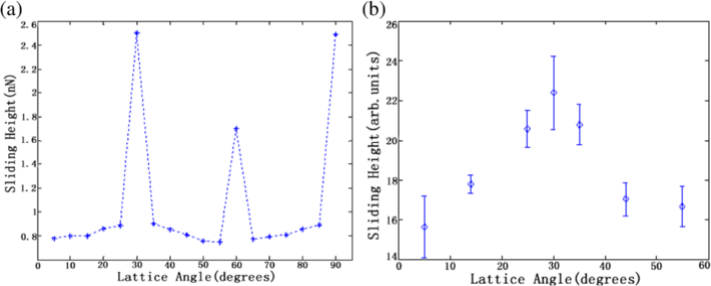
Sliding height as a function of lattice angles of graphene. Lattice angle 0° is the zigzag orientation. **a** Simulation results (from 5° to 90°). **b** Experimental results (5°, 14°, 25°, 30°, 35°, 44°, and 55°) obtained by the same tip and scan rate

Friction force signals vary with lattice orientations, as shown in Fig. 1. The lattice angles are 0°, 5°, 14°, 25°, 30°, 35°, 44°, 49°, and 55°, respectively. The friction force signal along zigzag orientation (lattice angle = 0°) has one peak period, and the signal along armchair orientation (lattice angle = 30°) has two-peak period. However, the period of the lattice angles between zigzag and armchair orientations is not clear. Therefore, the relationship between the frequency and lattice orientation has been further investigated based on wavelet transforms. The sliding height is plotted as a function of lattice angles in Fig. 2. From the simulation chart shown in Fig. 2a, a large increase of the sliding height at armchair and zigzag orientations is observed, suggesting more energy will be needed when the tip moves along these two orientations. Additionally, more energy along armchair orientations is needed than zigzag orientations. Figure 2b shows the experimental results having the same trend with simulation results.

**Fig. 3:**
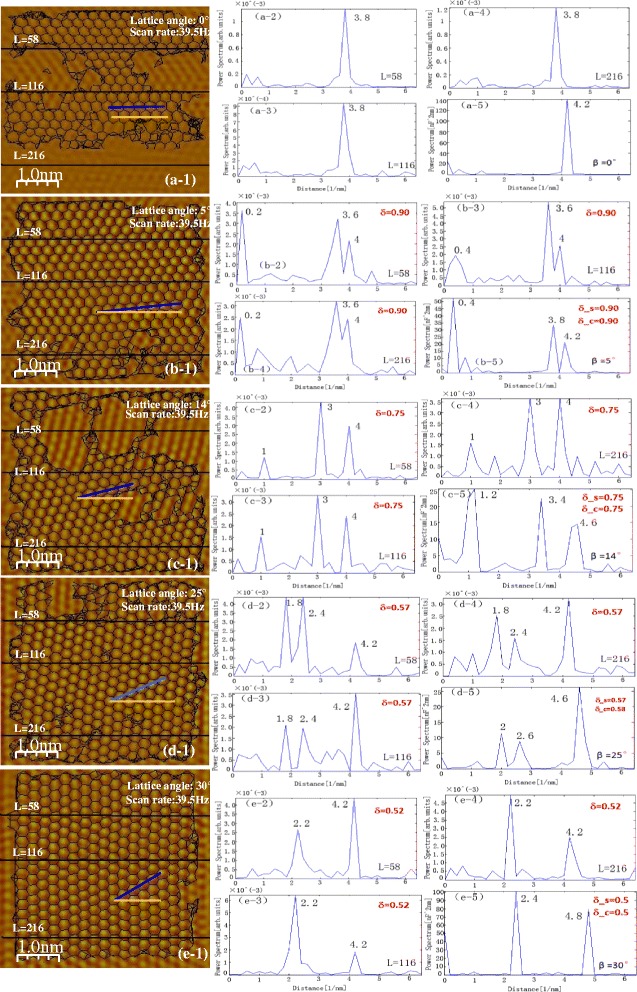
**a**–**d** Experiment versus simulation frequency power spectrum based on wavelet transform. *(x-1)*–*(x-4)* are experimental results, and L = 58, 116, and 216 indicate three single scan lines to be calculated in *(x-1)*, respectively. *(x-5)* are simulation results. *δ_s* and *δ_c* denote the ratio from the simulation and calculation (Eq. 2), respectively. *(x-1)* are “atomic resolution” AFM lateral force images with real lattice structures (*black mesh*)

The relationship between the frequency and lattice orientation has been further investigated based on wavelet transform. The simulation and experimental spatial frequency power spectrums based on wavelet transform are depicted in Fig. 3. The lattice angles are 0°, 25°, 30°, and 55°, respectively (The results of other angles see Additional file Figure S5). Three single experimental scanning signals (*L* = 58, 116, and 216) are calculated respectively. Figure 3 (x-1) is not true atomic resolution images. They are atomic corrugations from collective motions of multiple atoms on the tip. Therefore, the black mesh in the figure shows the real graphene lattice structures. According to the geometric relationship between the pseudo-lattice structure and the real lattice structure (see Ref. [35]), the real lattice structure can be automatically generated by programming using Matlab. Some fuzzy areas have no mesh. The experimental results correspond well with the simulation results. Different lattice orientations have different distributions of frequency power spectrum: one-peak, two-peak, and three-peak distribution receptivity. Although the main peak frequency is with less error, it cannot be used as a factor to determine the orientation. For lattice angle = 5° and 14°, both of the experimental main peak frequency is 4. The simulation main peak frequency is 4.6 with lattice angle = 14° and 25°, but the ratio values are much different. Therefore, the ratio *δ* of the two main peaks varying with lattice angles means δ can be used as a main factor to determine lattice orientations. These results will ultimately provide the platform for the development of graphene-based nanodevice.

To identify graphene lattice orientations, we propose a model based on the frequency ratio. The interaction potential of graphene used here is the same as the one of graphite [35]:

1 $$ {V}_{\mathrm{graphene}}\left({x}_{\mathrm{t}},{y}_{\mathrm{t}}\right)=-{V}_0\left[2 \cos \left(\frac{2\pi }{a}{x}_{\mathrm{t}}\right) \cos \left(\frac{2\pi }{a\sqrt{3}}{y}_{\mathrm{t}}\right)+ \cos \left(\frac{4\pi }{a\sqrt{3}}{y}_{\mathrm{t}}\right)\right] $$

where *V*_0_ is the barrier potential constant, *a* = 0.246 nm is the lattice constant of graphene, and (*x*_t_, *y*_t_) denotes the position of the tip.

Therefore, zigzag orientations (single peak) and armchair orientations (double peaks) are easily determined only by peak distributions of the frequency power spectrum from a single friction scan line. For the lattice angles between zigzags and armchairs, frequency ratio *δ* = *ω*_2/*ω*_1 (0° ≤ *θ* ≤ 30°) is defined, and then the lattice angles can be determined by

2 $$ \tan \theta =\sqrt{3}\left(1-\delta \right)\left(1+\delta \right),{0}^{\circ}\le \theta \le {30}^{\circ } $$

**Fig. 4:**
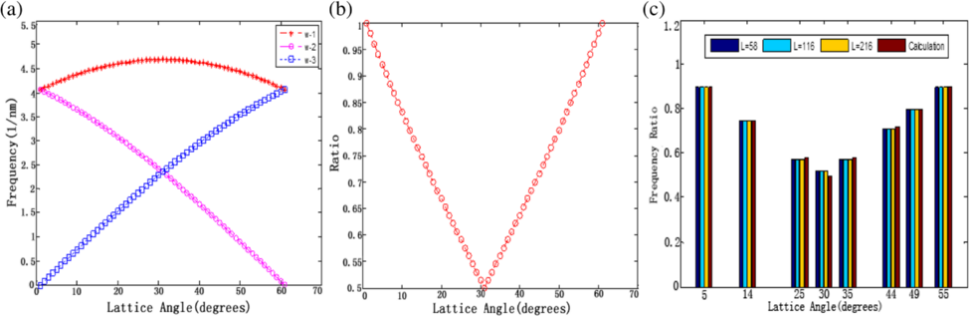
**a** Spatial frequencies as a function of the lattice angles of graphene. *Red line* indicates *ω*_1, *purple line* shows *ω*_2, and *blue line* indicates *ω*_3. **b** The frequency ratio *δ* as a function of a hexagonal periodicity of grapheme (simulation results). **c** The frequency ratio *δ* as a function of lattice angles. *L* = 58, 116, and 216 indicate the experimental single friction signal from the line of 58, 116, and 216, respectively. The *red bar* shows the calculation value computed from Eq. (2)

However, lattice angle cannot only be obtained by Eq. (2) in the real measurement. This is because *δ* is symmetrical at 30° in a hexagonal periodicity as shown in Fig. 4b, c. Therefore, the real lattice angle should observe the following rules:

1. Rotation (30°-*θ*) by anticlockwise, and scan a single line to calculate *δ*.
2. If this *δ* shows the armchair direction, then the calculated *θ* (0° < *θ* < 30°) should be the real lattice angle.
3. Otherwise, the real lattice angle should be (60°-*θ*).

**Fig. 5:**
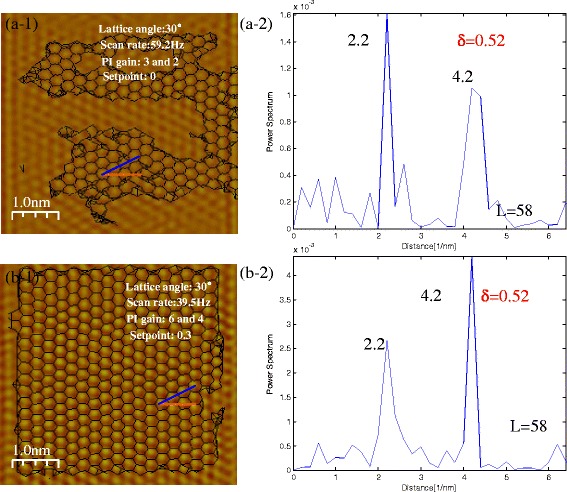
*δ* at different scan rate for the same lattice orientation (lattice angle: 30°). *(a-1)* and *(b-1)* are “Atomic resolution” AFM lateral force images (filtered by FFT) with lattice structures. The *blue line* shows 0° direction (zigzag direction), and the *yellow line* shows the scan direction. *(a-2)* and *(b-2)* are spatial frequency power spectrum. *δ* is the spatial frequency ratio of the scan line *L* = 58

In generally, the parameters such as scan rates, set points, proportional gain (P gain), and integral gain (I gain) have to be adjusted to obtain a good AFM image. Figure 5 shows the experimental results at the same lattice orientation with different scanning parameters. Figure 5(a-1) is an atomic resolution image obtained at scan rate = 59.2 Hz, P gain = 3, I gain = 2, and set point = 0. The black lines show the real atomic structure, which indicates the lattice angle is 14°. Figure 5(a-2) is the spatial frequency power spectrum which shows *δ* = 0.52. Figure 5(b-1) is the other atomic resolution image obtained with the same lattice angle at scan rate = 39.5 Hz, P gain = 6, I gain = 4, and set point = 0.3. Figure 5(b-2) shows the same distribution as Figure 5(a-2). Another experimental result is shown in Additional file 1: Figure S4. As observed, although scanning parameters are different, the spatial frequency power spectrums are the same at the same lattice angles.

**Fig. 6:**
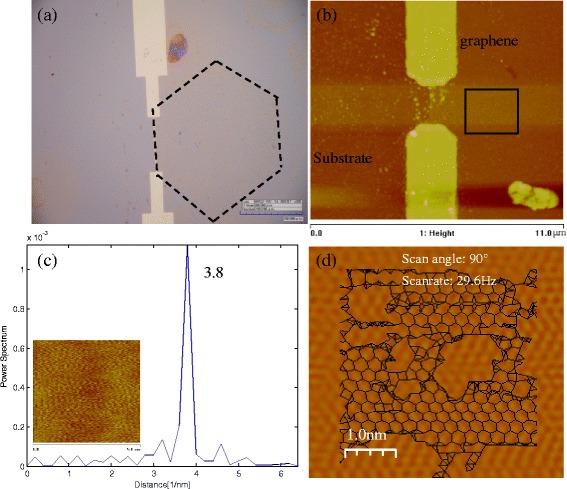
Lattice orientation identification on a monolayer graphene. **a** Optical image of the graphene. The *dot line* indicates the graphene sheet. *Blue scale bar*: 200 μm. **b** The topographic image of the graphene sheet. **c** Spatial frequency power spectrum from a single friction scanning line. The *inset* is a normal friction image from which lattice structure cannot be identified. **d** “Atomic image” of the spot indicated by a *black rectangle* in **b**

The lattice orientation determination of a monolayer graphene is demonstrated in Fig. 6. Using our method, there is no need to obtain an atomic resolution image. Even from an ordinary friction picture, the lattice orientation can be identified. Graphene was transformed to an electrode chip, which can be used as a marker as shown in Fig. 6a. A normal friction image (the inset of Fig. 6c), scan angle is 90°) was obtained on the graphene sheet (scanning spot shown in Fig. 6b by a black rectangle), from which the lattice orientation cannot be directly determined. However, the spatial frequency spectrum (Fig. 6c) of a single scanning line shows one-peak distribution indicating the scanning line is along the zigzag orientation. Because the graphene sheet here is single crystal [36], then the edges of hexagonal graphene sheet are deduced to zigzag orientations easily. In order to prove the correction of the identification, an atomic resolution image (a black rectangle in Fig. 6b is indicating the scanning position, not the scanning area) was obtained in air under ambient conditions with several hours shown in Fig. 6d; the lattice orientation is consistent with our method’s result.

## Conclusions

The properties of graphene strongly rely on its edge structures. However, there is no rapid way to determine the lattice orientation on graphene. A simple and controllable method is developed to distinguish the lattice orientation of graphene appropriately. The method proposed in the manuscript only needs one or two scan lines to obtain the frequency ratio based on wavelet transform. Both theoretical and experimental results at the atomic scale have shown that the frequency ratios vary with the lattice orientations on graphene. In addition, an adjusted angle to the desired lattice orientation can be easily calculated based on the frequency ratio and the distribution obtained in this work, ultimately providing the platform for graphene engineering and graphene based nanodevices. In the future, the effects of the structural complexities of graphene on frequencies will be investigated, such as local strain, defects, and puckering. Recently, some studies [37–41] have shown that the structural complexities of 2D materials strongly influence friction forces. Therefore, they will influence the frequency also. These effects will be systematically studied in the next step.

## Additional file

Additional file 1: Supporting Information. (PDF 1 mb)

## References

[1] Novoselov KS, Geim AK, Morozov SV, Jiang D, Zhang Y, Dubonos SV (2004). Electric field effect in atomically thin carbon films. Science.

[2] Novoselov KS, Falko VI, Colombo L, Gellert PR, Schwab MG, Kim K (2012). A roadmap for graphene. Nature.

[3] Han SJ, Reddy D, Carpenter GD, Franklin AD, Jenkins KA (2012). Current saturation in submicrometer graphene transistors with thin gate dielectric: experiment, simulation, and theory. ACS Nano.

[4] Guom Z, Dong R, Chakraborty PS, Lourenco N, Palmer J (2013). Record maximum oscillation frequency in C-face epitaxial graphene transistors. Nano Lett.

[5] Han SJ, Garcia AV, Oida S, Jenkins KA, Haensch W (2014). Graphene radio frequency receiver integrated circuit. Nat Comm.

[6] Ghadiry M, Ismail R, Saeidmanesh M, Khaledian M, Manaf A (2014). Graphene nanoribbon field-effect transistor at high. Nanoscale Res Lett.

[7] Wu YQ, Lin YM, Bol AA, Jenkins KA, Xia F, Farmer DB (2011). High-frequency, scaled graphene transistors on diamond-like carbon. Nature.

[8] Polat EO, Kocabas C (2013). Broadband optical modulators based on graphene supercapacitors. Nano Lett.

[9] Karimi H, Yusof R, Rahmani R, Hosseinpour H, Ahmadi MT (2014). Development of solution-gated graphene transistor model for biosensors. Nanoscale Res Lett.

[10] Wang Y, Yang T, Lao J, Zhang R, Zhang Y, Zhu M (2015). Ultra-sensitive graphene strain sensor for sound signal acquisition and recognition. Nano Res.

[11] Wang Y, Yang R, Shi ZW, Zhang LC, Shi DX, Wang E, Zhang GY (2011). Super-elastic graphene ripples for flexible strain sensors. acs nano.

[12] Zhu WJ, Neumayer D, Perebeinos V, Avouris P (2010). Silicon nitride gate dielectrics and band gap engineering in graphene layers. Nano Lett.

[13] Kim M, Safron NS, Han E, Arnold MS, Gopalan P (2010). Fabrication and characterization of large-area, semiconducting nanoperforated graphene materials. Nano Lett.

[14] Ouyang FP, Peng S, Liu Z, Liu Z (2011). Bandgap opening in graphene antidot lattices: the missing half. ACS Nano.

[15] Feng J, Li WB, Qian XF, Qi JS, Qi L, Li J (2012). Patterning of graphene. Nanoscale.

[16] Datta SS, Strachan DR, Khamis SM, Johnson ATC (2008). Crystallographic etching of few-layer graphene. Nano Lett.

[17] Ci L, Xu Z, Wang L, Gao W, Ding F, Kevin FK, Boris IY, Pulickel MA (2008). Controlled nanocutting of graphene. Nano Res.

[18] Campos LC, Manfrinato VR, Sanchez-Yamagishi JD, Kong J, Jarillo-Herrero P (2009). Anisotropic etching and nanoribbon formation in single-layer graphene. Nano Lett.

[19] Gao L, Ren W, Liu B, Wu Z, Jiang C, Cheng HM (2009). Crystallographic tailoring of graphene by nonmetal SiOx nanoparticles. J. Am Chem Soc.

[20] Giesbers AJM, Zeitler U, Neubeck S (2008). Nanolithography and manipulation of graphene using an atomic force microscope. Sol St Comm.

[21] Tapaszto L, Dobrik G, Lambin P, Biro LP (2008). Tailoring the atomic structure of graphene nanoribbons by scanning tunnelling microscope lithography. Nat Nano.

[22] Weng L, Zhang LY, Chen YP, Rokhinson LP (2008). Atomic force microscope local oxidation nanolithography of graphene. Appl Phys Lett.

[23] Zhang Y, Liu L, Xi N, Wang Y, Dong Z, Wejinya UC (2011). Dielectrophoretic assembly and atomic force microscopy modification of reduced graphene oxide. J Appl Phys.

[24] Zhang Y, Liu L, Xi N, Wang Y, Dong Z, Wejinya UC (2012). Cutting forces related with lattice orientations of graphene using an atomic force microscopy based nanorobot. Appl Phys Lett.

[25] Zhang L, Diao S, Nie Y, Yan K, Liu N, Dai B (2011). Photocatalytic patterning and modification of graphene. J Am Chem Soc.

[26] Fischbein MD, Drndic M (2008). Electron beam nanosculpting of suspended graphene sheets. Appl Phys Lett.

[27] Bell DC, Lemme MC, Stern LA, Marcus CM (2009). Precision cutting and patterning of graphene with helium ions. Nanotechnology.

[28] Lemme MC, Bell DC, Williams JR (2009). Etching of graphene devices with a helium ion beam. ACS Nano.

[29] Naruo S, Hideaki O, Noriaki I, Kouji M (2010). Atomic-scale friction of monolayer graphenes with armchair- and zigzag-type edges during peeling process. e-J Surf Sci Nanotech.

[30] Hembacher S, Giessibl FJ, Mannhart J, Quate CF (2003). Revealing the hidden atom in graphite by low-temperature atomic force microscopy. PNAS.

[31] Lee CG, Li Q, Kalb W, Liu XZ, Berger H (2010). Frictional characteristics of atomically thin sheets. Science.

[32] Gao L, Ren W, Xu H, Jin L, Wang Z, Ma T (2012). Repeated growth and bubbling transfer of graphene with millimetre-size single-crystal grains using platinum. Nat Comm.

[33] Burrus CS, Gopinath RA, Guo H (1997). Introduction to wavelets and wavelet transforms.

[34] Ding W, Qin S, Miao L, Xi N, Li H (2012). The application of wavelet transform and wavelet lifting in signal processing of EGG. J Biomed Eng.

[35] Holscher H, Schwarz UD, Zwomer O, Wiesendanger R (1998). Consequences of the stick-slip movement for the scanning force microscopy imaging of graphite. Phys Rev B.

[36] Yu QK, Jauregui LA, Wu W, Colby R, Tian J, Su Z, Cao H (2011). Control and characterization of individual grains and grain boundaries in graphene grown by chemical vapour deposition. Nat Mater.

[37] Rastei MV, Heinrich B, Gallani JL (2013). Puckering stick-slip friction induced by a sliding nanoscale contact. Phys Rev Lett.

[38] Rastei MV, Heinrich B, Gallani JL (2014). Sliding speed-induced nanoscale friction mosaicity at the graphite surface. Phys Rev B.

[39] Choi JS, Kim JS, Byun IS, Lee DH, Lee MJ, Park BH (2011). Friction anisotropy-driven domain imaging on exfoliated monolayer graphene. Science.

[40] Boland MJ, Nasseri M, Hunley DP, Ansary A, Strachan DR (2015). Striped nanoscale friction and edge rigidity of MoS2 layers. RSC Adv.

[41] Gallagher P, Lee M, Amet F, Maksymovych P, Wang J, Wang S, et al. One-dimensional ripple superlattices in graphene and hexagonal boron nitride. 2015; arXiv preprint arXiv: 1504.05253.

