# Supplementary material for: Online Determination of Graphene Lattice Orientation Through Lateral Forces
Source: Nanoscale Res Lett. 2016 Aug 2;11:353. doi: 10.1186/s11671-016-1553-z (PMC4971012; doi:10.1186/s11671-016-1553-z)
Supplement: Additional file 1: — Supporting Information. (PDF 1 mb) [file 11671_2016_1553_MOESM1_ESM.pdf]

Supporting Information

On-Line Determination of Graphene Lattice Orientation though Lateral Forces

Yu Zhang,[1,2] Fanhua Yu,[1] Guangyong Li, [2,3] Lianqing Liu,[2*] Guangjie Liu,[1] Zhiyong Zhang,[1] Yuechao Wang, [2] Uchechukwu C. Wejinya[2,4] and Ning Xi, [2,5*]

[1]*Department of Computer Science and Technology, Changchun Normal University, Changchun, 130032,China*

[2]*State Key Laboratory of Robotics, Shenyang Institute of Automation Chinese Academy of Sciences, Shenyang, 110016, China*

[3]*Department of Electrical and Computer Engineering, University of Pittsburgh, Pittsburgh, 15261, USA*

[4]*Department of Mechanical Engineering, University of Arkansas, Fayetteville, Arkansas, 72701,USA*

[5] *Emerging Technologies Institute, The University of Hong Kong, Hong Kong, China*

* Correspondence: lqliu@sia.cn; xining@hku.hk

1. Atomic imaging of graphene and friction signals with different lattice orientations

Figure S1(a) shows the graphene sample for atomic imaging. The black rectangle indicates the scanning site. The thicknesses of graphene are around 2.367nm, 2.455nm and 2.226nm, respectively, as shown in Fig. S1(a-1), (b-1) and (c-1). Figure S1(a-2), (b-2)–(b-8) and (c-2) displays a series of atomic resolution AFM friction images of graphene obtained under ambient conditions at different scan rates(shown in the left top corner). The insets are raw images as shown in the right bottom corner. The real lattice structures are shown on the FFT filtered images. The blue line shows 0° direction (zigzag direction) and the yellow line shows the scan direction. Then the measured lattice angles are 0°, 5°, 14°, 25°, 30°, 35°, 44°, 49° and 55°, respectively. The corresponding single friction force signals are shown in Fig. S2, which shows that the experimental results are consistent with the simulation results.

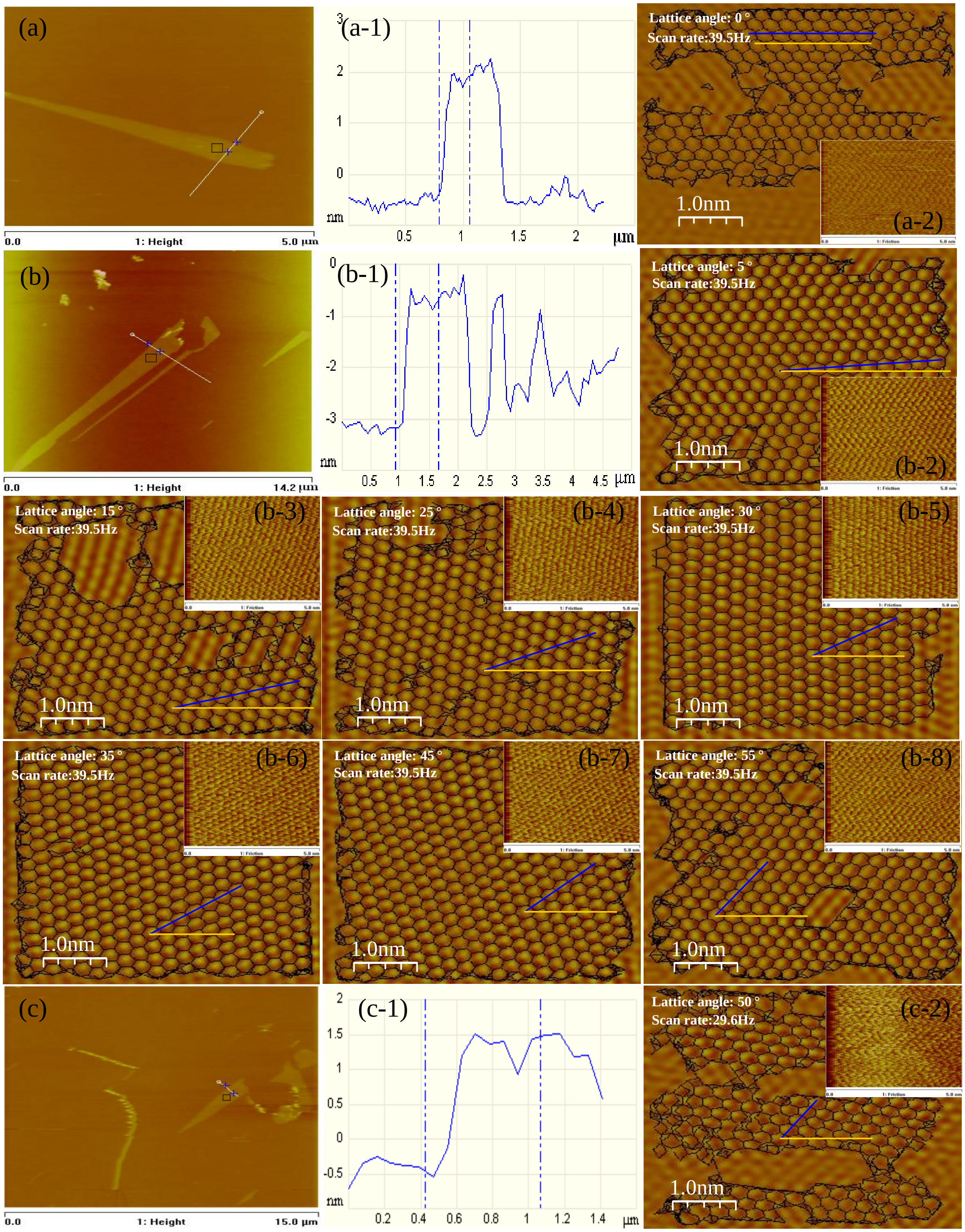

FIG. S1. AFM images of graphene. (a) (b)(c)The topographic images of graphene on Si/SiO2 substrate. The black rectangle indicates the scanning site for atomic imaging. (a-1)(b-1)(c-1) Height analysis trace,showing the thickness of graphene (while line indicating) in (a) ,(b) and (c) are around2.367nm,2.455nm and 2.226nm, respectively. (a-2),(b-2) –(b-8),(c-2) The "atomic resolution" AFM lateral force images (filtered by FFT) with lattice structures in ambient conditions with different lattice angles (shown in the left top corner), respectively. The raw images are shown inset. The blue line shows 0° direction (zigzag direction) and the yellow line shows the scan direction.

2. Theoretical Modeling for atomic friction behavior

A two-dimensional Tomlinson model is used to study the atomic friction behavior on graphene. In this model, the AFM tip is handled as a point mass which is coupled to a support *M* ('AFM cantilever') by springs and the support *M* moves along the*x* direction with a constant velocity v_M. The interaction between the tip and the surface is described by a periodic potential $V(x_t, y_t)$. All energy dissipation is considered by a simple velocity-dependent damping term γ.

The motion of the tip in the interaction potential is given by[1]

$$m_x \ddot{x}_t = k_x(x_M - x_t) - \frac{\partial V(x_t, y_t)}{\partial x_t} - \gamma_x \dot{x}_t$$
$$m_y \ddot{y}_t = k_y(y_M - y_t) - \frac{\partial V(x_t, y_t)}{\partial y_t} - \gamma_y \dot{y}_t \quad (1)$$

where(x_t, y_t),(x_M, y_M)are the position of the tip and *M*, respectively. m_x and m_y are the effective masses of the system, k_x and $k_y$the spring constants, and γ_x and γ_x are the dampingconstants. The path of the tip $(x_t(t), y_t(t))$ on the sample surface can be calculated from equation (1), and then the lateral forces $F_x = k_x(x_M - x_t)$ and $F_y = k_y(y_M - y_t)$ can be obtained. In the experiments, the lateral force is measured only in the scanning direction, therefore, only the force acting on the support in the *x* direction is considered as the friction force.

The interaction potential of graphene used here is the same as the one of graphite [1]:

$$V_{\text{graphene}}(x_t, y_t) = -V_0[2\cos(\frac{2\pi}{a}x_t)\cos(\frac{2\pi}{a\sqrt{3}}y_t) + \cos(\frac{4\pi}{a\sqrt{3}}y_t)] \quad (2)$$

where $a = 0.246$ nm. The lattice structure can be rotated by a simple coordinate transformation, whereas the [100] direction (zigzag orientation) is defined as lattice angle 0 °.

The equation (1) is solved using a 4th order Runge-Kutta method with the following initial conditions.

$$x(0) = 0, \dot{x}(0) = 0, \mathrm{y}(0) = 0, \dot{\mathrm{y}}(0) = 0 \quad (3)$$

To obtain a numericalsolutiontoequation (1), suitable parameters must to be found. However, the exact values of these parameterscannot be obtained from experiments directly and these parameters have to be estimated. All the numerical calculations presented here are obtained using the set of parameters in Ref.[1]: $V_0 = 0.5\text{eV}, k_x = k_y = 25\ \text{N/m}$ (which are

typically used for AFM experiments), $m_x = m_y = 10^{-8}\,\mathrm{kg}$ (with these effective masses, the system has a common resonance frequency of $1/(2\pi)\sqrt{k_x/m_x} \approx 8\mathrm{kHz}$), $\gamma_x = \gamma_y = 10^{-3}\,\mathrm{Ns/m} \approx 2\sqrt{k_x m_x}$ (critical damping), and $v_M = 40\,\mathrm{nm/s}$ (a commonly used scanning velocity).

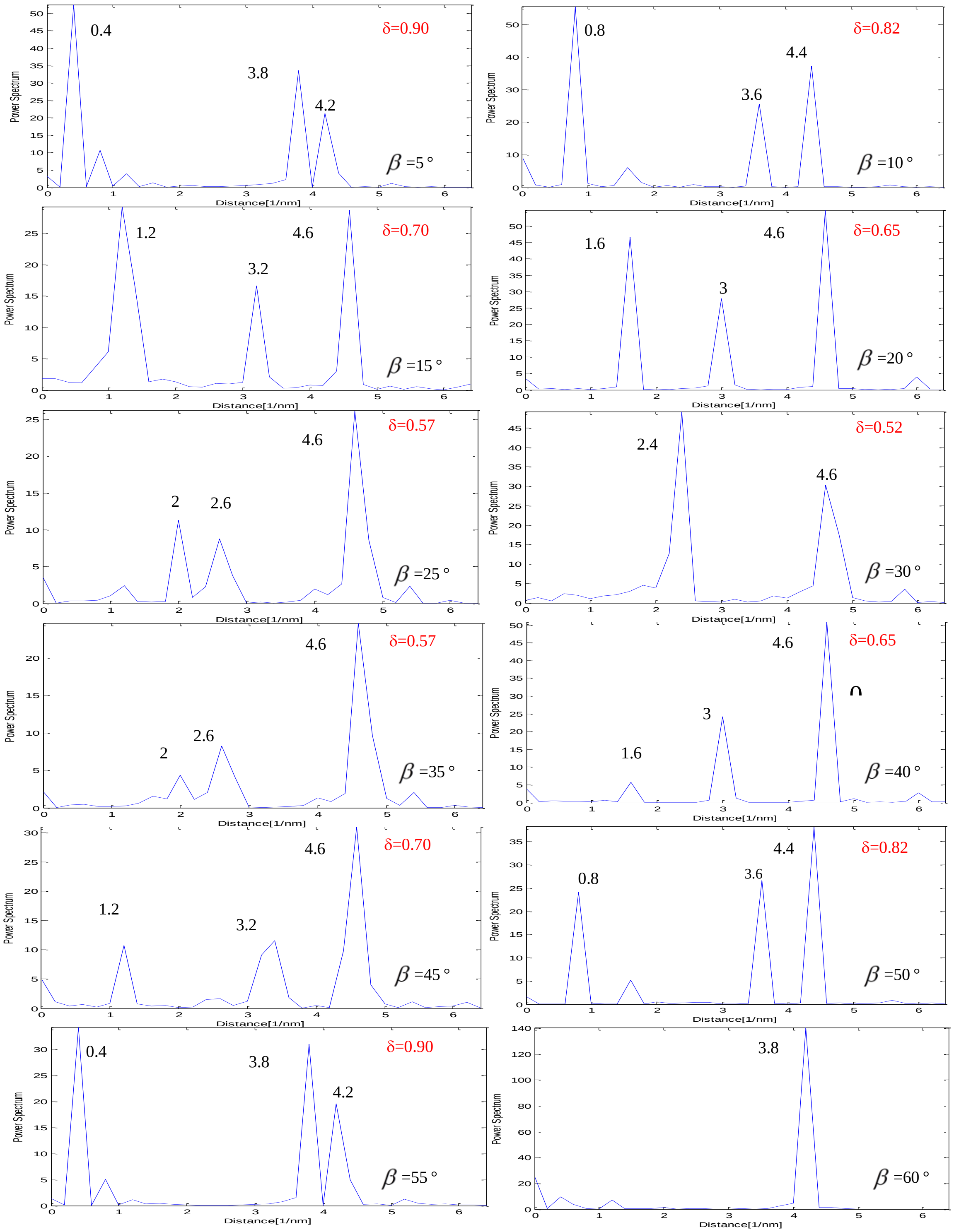

FIG. S2. Simulation frequency power spectrums of different lattice orientations. β =0 °is defined as the zigzag orientation. β =30 °indicate armchair orientation. δ is the spatial frequency ratio.

3. Simulation spatial frequency power spectrums of different lattice orientations

Figure S2 shows the simulation spatial frequency power spectrums of friction force signals at different lattice angles. The simulation friction force signals were obtained from two-dimensional Tomlinson model depicted above. The lattice angle β=0 ° is defined as the zigzag orientation, thusβ=30 °indicate armchair orientations. δ is the spatial frequency ratio of two main peaks of the spectrums. The results show that the frequency power spectrum distributions vary with different lattice orientations. There are three types of distributions: one peak, two peaks and three peaks. The zigzag orientation has one-peak distribution; the armchair orientation has double-peak distribution. The angles in between with these two orientations have three-peak distribution, but the positions of the peaks are shifted with different orientations. Therefore, the frequency ratio δvaries with lattice orientations.

4. Wavelet transforms

Wavelet transforms include continuous wavelet transform (CWT) and discrete wavelet transform (DWT). The CWT of a signal *x(t)* is defined as[2]:

$$WT_x(a,\tau) = \frac{1}{\sqrt{a}} \int_{-\infty}^{+\infty} x(t)\psi^*(\frac{t-\tau}{a})dt$$

where *x(t)* is the sample signal, *ψ*()* is the wavelet basis function.

The simulation and experimental friction force signals are all computed by DWT . DWT with respect to the wavelet $\psi(t)$ could be represented as follows[2]:

$$CWT_x(iT_s,a) = T_s \frac{1}{\sqrt{a}} \sum_n x(nT_s)\psi^*(\frac{(n-i)T_s}{a})$$

where *x()* is the sample signal, T_s is the sampling interval, and *i* is the integer sample number.

Based on the four-step wavelet decomposition, a single friction force signal (SFFS) decomposition figure is plotted in Fig. S3. The original signal is denoted by x, and d1, d2, d3, d4 and a4 are the corresponding four-step decomposition signal, respectively.

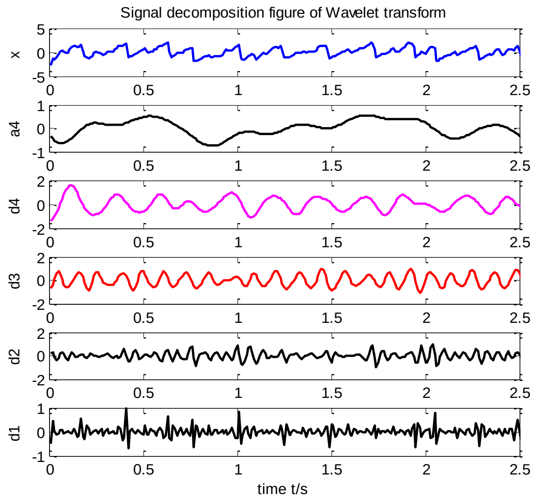

FIG. S3. Signal decomposition figure of SFFS based on wavelet transform. X is the original signal.

5. The effect of scanning parameters on the frequency ratio

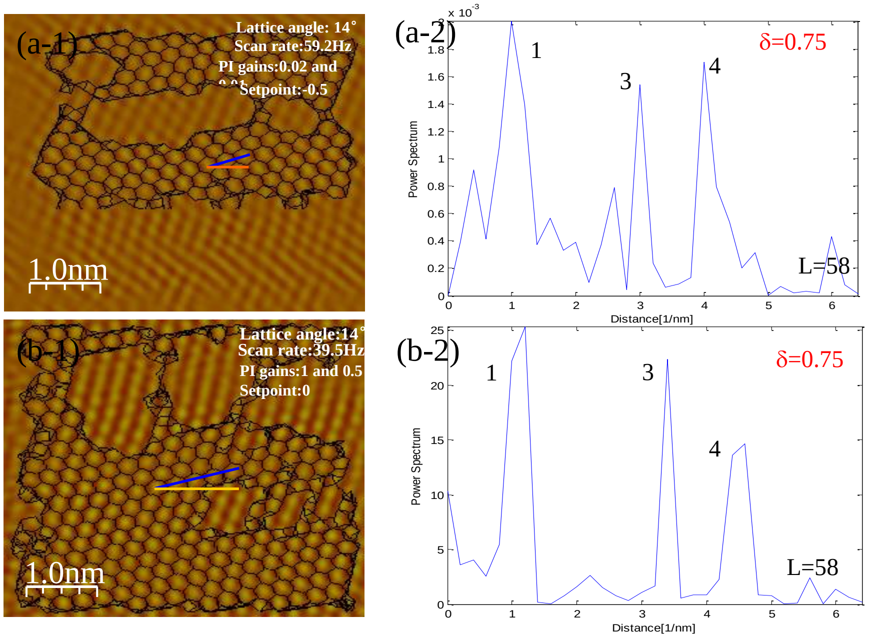

FIG. S4. δ at different scan rate for the same lattice orientation (lattice angle:14°). (a-1),(b-1) "Atomic resolution" AFM lateral force images (filtered by FFT) with lattice structures. The blue line shows 0° direction (zigzag direction) and the yellow line shows the scan direction. (a-2), (b-2) spatial frequency power spectrum. δ is the spatial frequency ratio of the scan line L=58.

6. Frequency anisotropy of graphene based on a wavelet transforms

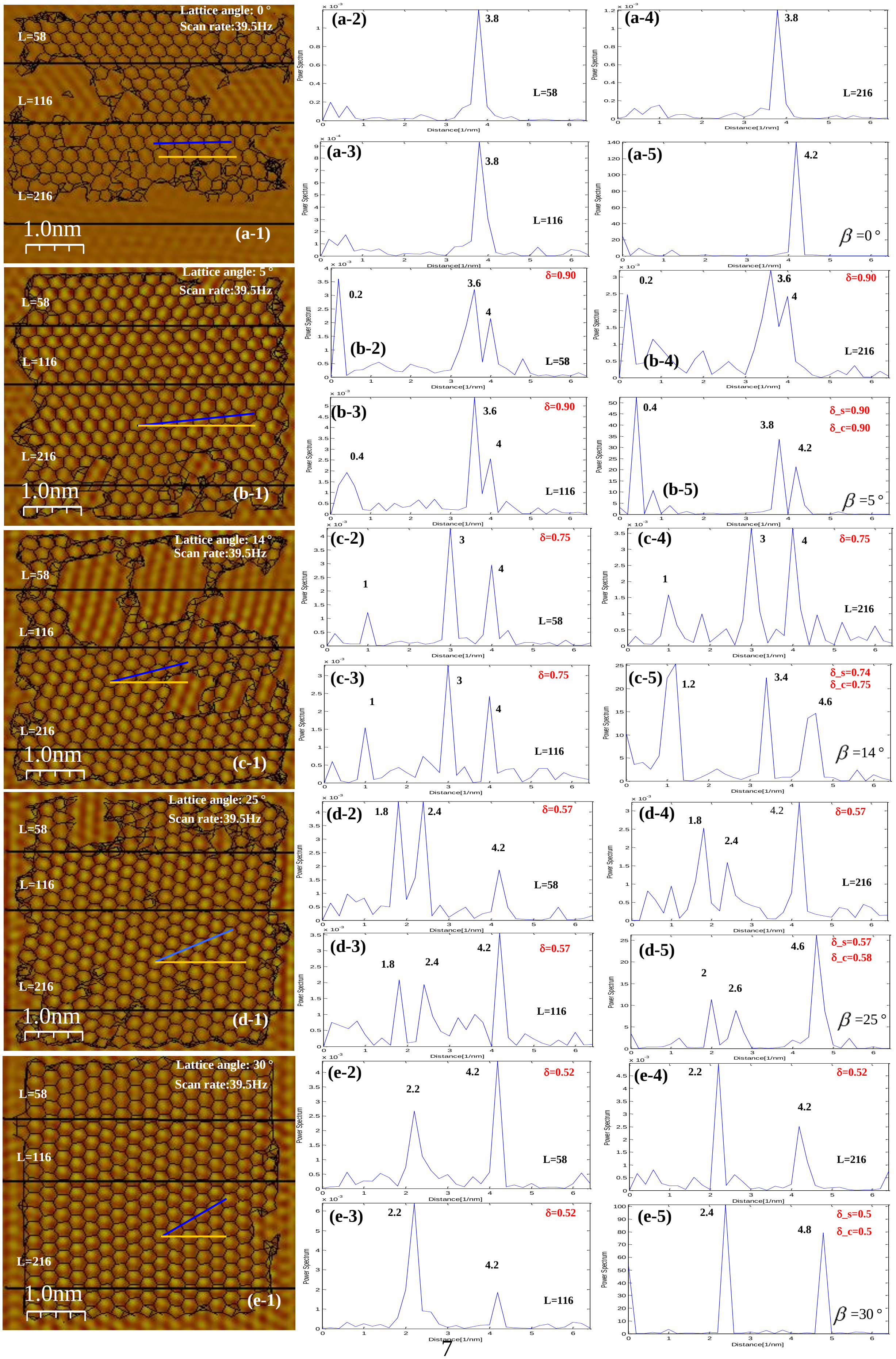
Lattice angle: 0 °
Scan rate:39.5Hz
L=58
L=116
L=216
1.0nm
(a-1)
(a-2)
3.8
L=58
(a-3)
3.8
L=116
(a-4)
3.8
L=216
(a-5)
4.2
β =0 °
Lattice angle: 5 °
Scan rate:39.5Hz
L=58
L=116
L=216
1.0nm
(b-1)
(b-2)
0.2
3.6
4
δ=0.90
L=58
(b-3)
0.4
3.6
4
δ=0.90
L=116
(b-4)
0.2
3.6
4
δ=0.90
L=216
(b-5)
0.4
3.8
4.2
δ_s=0.90
δ_c=0.90
β =5 °
Lattice angle: 14 °
Scan rate:39.5Hz
L=58
L=116
L=216
1.0nm
(c-1)
(c-2)
1
3
4
δ=0.75
L=58
(c-3)
1
3
4
δ=0.75
L=116
(c-4)
1
3
4
δ=0.75
L=216
(c-5)
1.2
3.4
4.6
δ_s=0.74
δ_c=0.75
β =14 °
Lattice angle: 25 °
Scan rate:39.5Hz
L=58
L=116
L=216
1.0nm
(d-1)
(d-2)
1.8
2.4
4.2
δ=0.57
L=58
(d-3)
1.8
2.4
4.2
δ=0.57
L=116
(d-4)
1.8
2.4
4.2
δ=0.57
L=216
(d-5)
2
2.6
4.6
δ_s=0.57
δ_c=0.58
β =25 °
Lattice angle: 30 °
Scan rate:39.5Hz
L=58
L=116
L=216
1.0nm
(e-1)
(e-2)
2.2
4.2
δ=0.52
L=58
(e-3)
2.2
4.2
δ=0.52
L=116
(e-4)
2.2
4.2
δ=0.52
L=216
(e-5)
2.4
4.8
δ_s=0.5
δ_c=0.5
β =30 °
Power Spectrum
Distance[1/nm]

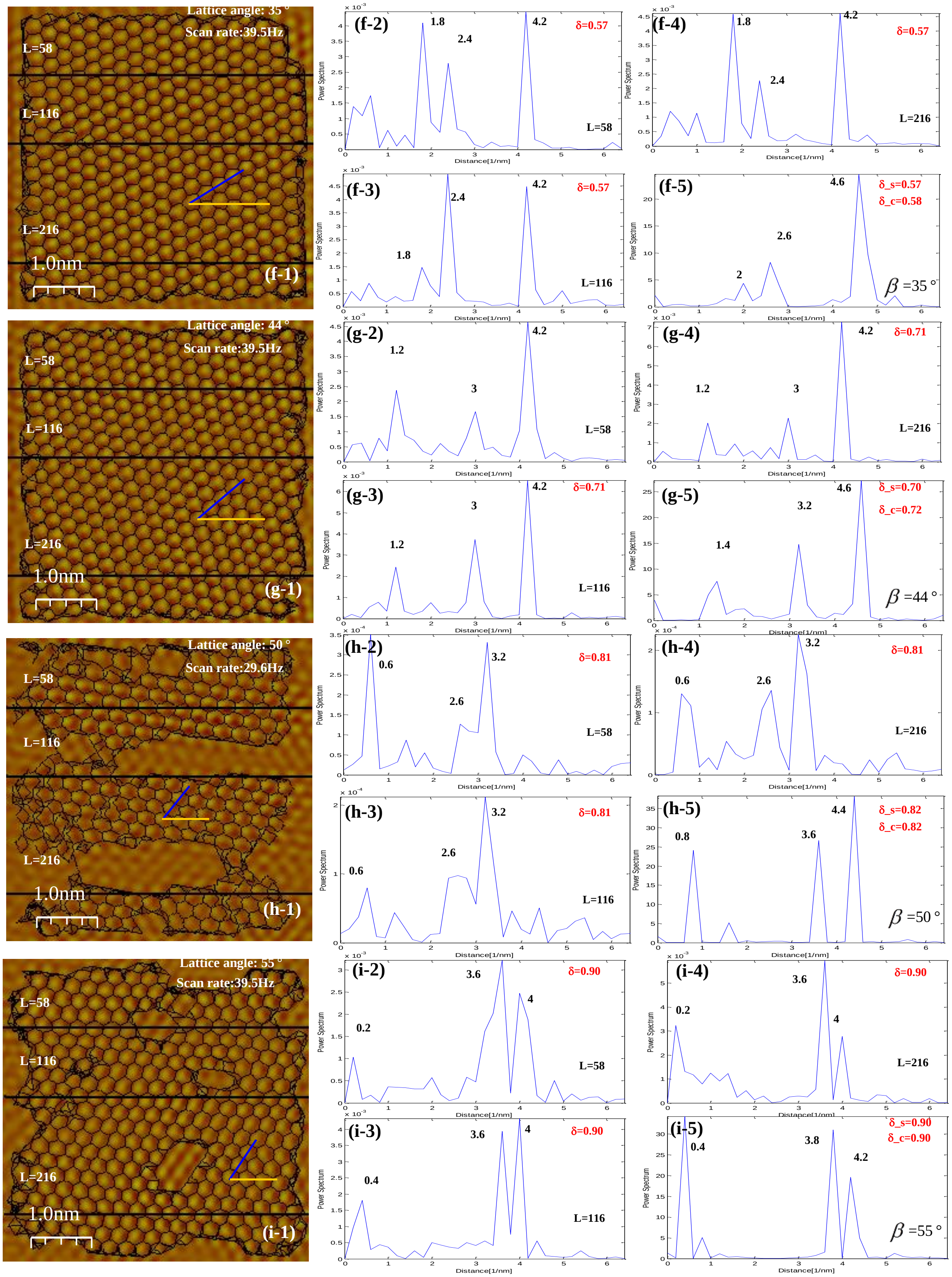

FIG. S5. Experiment versus simulation spatial frequency power spectrum based on wavelet transform. (x-1)-(x-4) are experimental results, and L=58,116 and 216 indicate three single scan lines to be calculated in (x-1), respectively. (x-5) are simulation results.δ_s and δ_c denote

the ratio from the simulation and calculation (Eq.4), respectively. (x-1) are “atomic resolution” AFM lateral force images with real lattice structures (black mesh)

[1] Holscher, H., Schwarz, U.D., Zwomer, O. & Wiesendanger, R. Condequences of the stick-slip movement for the scanning force microscopy imaging of graphite. *Physical Review B.*, **57**, 2477-2481(1998)

[2] Ding, W., Qin, S., Miao, L., Xi, N., & Li, H. The Application of Wavelet Transform and Wavelet Lifting in Signal Processing of EGG. Journal of biomedical engineering, 29(4), 745-749(2012).
